# Gathering and Distributing the News

**Published:** 1884-07

**Authors:** Ausburn Towner


### GATHERING AND DISTRIBUTING THE NEWS.

AUSBURN TOWNER.

Away up in the eighth story of the Western Union Telegraph company's building at Broadway and Dey streets, New York, is the heart of a system, whose pulse beats, it is no hyperbole to say, can be felt on every habitable spot on the globe, and in some spots too that are not habitable. It is the headquarters of the Associated Press of New York. Its likeness to a heart is borne out from the fact that the Western Union wires, like veins, keep it in constant and live communication with the extremities of the earth, and it furnishes the life that makes the whole system possible.

It is an old story of how the seven most conspicuous newspapers in New York city, years ago, banded together and left it to one man and his assistants, to gather for them from all quarters, the incidents and happenings of the day, reducing for them, their expenses in that department of journalism, and yet leaving them able to publish in full whatever of interest was transpiring anywhere. Something of the growth and extent of this system is also more or less known, and the fact that anywhere, any incident of interest or value always gets sent into the Associated Press and is always welcome. The practical, every-day working of the system is but little known, and there is so much of it,—it has so many ramifications; so many ins and outs, it spreads over so vast a territory that a book could easily be written about it and then not exhaust the topic or relate half the triumphs and successes that it has everywhere met with and is continually meeting.

The few men in the headquarters office form only what might be called the staff of the general-in-chief of the immense army of persons scattered all over the world who look out for the interests of the concern, and in the headquarters office there is no cessation of work. The day men come on duty almost immediately after the night men have put on their overcoats and gone home, and the night men appear again before the day men have left their desks. There is an uninterrupted clicking of telegraph instruments the twenty-four hours around, even as life and trouble and crime is exhuberant, or soul disturbing or rampant somewhere on the globe every minute of the twenty-four hours of the day.

The New York City Associated Press, the mother of all similar organizations of the kind in this country, is the center around which they all circulate and to which they all look, and in these headquarters are established agents for them all; the New England Press has a representative; the Western Press; the Southern, California and the New York State Press, and to all of these, by contract, the Associated Press furnishes all the matter it receives. Every line that comes into the office is manifolded, that is, by means of thin paper and paper that is prepared, one writer can make at one time a great number of copies of any message, and one of these copies is sent to each member of the Associated Press, and each agent of the other Presses. Of course, no one paper unless very large, could use all the matter transmitted, for some days there are more than 100,000 words sent in, and all of the matter is not suitable or interesting for every locality. The New England Press man would throw away what would be of interest to the Western Press, or the Southern man would not use what would be just the thing for the California papers. Yet the whole news of the day comes in and the different representatives must read it all over carefully lest an item sensational or important should be overlooked. One can judge somewhat of the labor necessary by imagining himself obliged to read every word of a magazine like Harper or the Century, every night, or every day, to make selections from. After having decided what is news for their localities, the agents must then edit it. Some who have no care for the amount they telegraph, prepare their matter to go immediately into the hands of the printer on its receipt, being careful of their expressions and seeing that all of the particles and small words are correctly inserted; others who have no discretion send their selections just as they are brought into them, while others, limited as to the number of words, yet anxious to cover the whole field of news, compress and condense, cut out words that will still leave the sense perfect, and put a whole paragraph into two or three lines. As an example of this latter, the following item went over the wires in this shape:

WASHINGTON.—Commander Schley, charge Greeley expedition in letter Secretary Navy, suggests "Bear" dispatched from New York, April 25. "Thetis" follow May 1. "Alert" not later May 10. Suggestions and recommendations adopted department."

Under experienced manipulation of the telegraph editor of the newspaper, the dispatch appears, properly dated:

WASHINGTON.—Commander W. T. Schley who has charge of the Greeley relief expedition, in a letter to the Secretary of the Navy, suggests that the "Bear" be dispatched from New York on April 25th. The "Thetis" should follow by May 1st, and the "Alert" not later than May 10th. His suggestions and recommendations have been adopted by department."

It will be seen that in comparing the two paragraphs, just thirty words were saved in the transmission, and yet the complete sense was retained to one who keeps track of events. It leads one to the observation that there are a great many unnecessary words used in the English language. Any one can satisfy himself of this by taking up any book at random and re-constructing any of the sentences upon which his eye may rest. Elegancies and rhetorical display may vanish, but the sense, if there is any there, will remain. A small paragraph like the one given is mere play, but when it becomes necessary to reduce thirty or forty pages to as many lines and yet retain their meat and juice, that is what starts the perspiration. Yet it is done and satisfactorily done too every day; so that one who reads may daily keep abreast with everything going on in the world, and yet not be obliged to wade through columns of matter to get at it. It is the modern way of conducting a newspaper.

On the other hand, I have known the California man to send 2,000 words, or fully a column of the Elmira *Gazette* on one subject without erasing an a or a the. The California people have the same advantage over us that we have over the London papers, the difference in time giving their man here an opportunity to "crib" all he wants from the New York morning papers, and send it on to them long before their hour of going to press. One of their night men told me recently that he had sent matter from New York as late as nine o'clock in the morning, which appeared in the city edition of the morning San Francisco papers. It is a matter of nightly occurrence to get London dispatches dated the next day, containing extracts from the *Times* or *Telegraph*, and most of the night Suakim and Cairo news is of that which at the time of its receipt, is our to morrow.

Each of the agents I have named, has a telegraph instrument and operator right at his side and all to himself. With some of the Presses the wire used is called a leased one: that is the Press has a contract with the Western Union, by which, for a certain sum, an exclusive wire is furnished them, for specified hours. This has no regard to the number of words or amount of matter sent. Others pay a certain rate per word for the transmission of their matter. The sending is all done at once to each of the members of the Association; the operator here calling each office and on getting a response from all, starting in with his report, as for instance on the State Press, he calls up the operators at the dozen or fifteen points where the report is taken; the circuit is complete from here around through Buffalo to Bradford, Pa., and by the way of Syracuse to Binghamton and Elmira, the operators at the several points write out their several reports and send them to the newspapers to which they belong. I care not how accustomed a man may become to the wonders of this century, or whether he has been born in the midst of them and they are so a part of his every-day life, there is something to make a thoughtful man stop every now and then with astonishment. One sitting here in his office can at one and the same instant virtually talk with persons in localities so widely separated from him and as well, from each other. Our own state, in this respect, seems small, when we consider the western circuit reaching to Milwaukee and St. Paul, or the southern circuit accomplishing the same result by Charleston and New Orleans, taking in on its way, Jacksonville, Florida, and Mobile.

One unacquainted with the practical working of the Associated Press, might naturally inquire how it was that it is constantly in receipt of news from everywhere. How is it possible to arrange for such an immense sweep. In some prominent points like Boston, Washington, and London, England, there are regularly salaried agents, sometimes with assistants whose duties are to forward everything of interest that may be gleaned anywhere in their territory. Mostly, in our own country and in our own state especially, the agreement, or a part of the agreement entered into by the several associations is that they shall furnish to the Associated Press, news of their several localitities in exchange for the news they receive. I heard once, a person, not much of a newspaper man, liken this part of the contract to a couple of village gossips, who got regularly together at stated periods, to "swap lies," as he called it. It is part of the agreement for instance with the State Press, that the Elmira *Advertiser*, being a part of that Association, shall furnish to the Associated Press news from its locality, as it is that the Rochester *Democrat and Chronicle*, or the Schenectady *Union*, shall do the same by their several localities. Those persons sending the news are not necessarily paid agents of the Associated Press, are oftener not; and what they do, enures to the benefit of the newspaper with which they are connected in helping to carry out the contract of their association.

In the wider sweep of gathering intelligence, similar processes of exchanging news is carried on from distributing points, Cincinnati is a centre for the whole south-west and far west, and Chicago for the north-west. They cover each its separate territory as the New York Press covers it, and whatever comes to them is transmitted to New York, the great hopper of the eternal grist whose grinding never ceases.

This will explain to an observer how the Associated Press covers so vast a territory, and how local papers are able, if they are fortunate enough to be members of the enormous system, are able to spread before their readers on one morning, events that have happened the evening before at the distance of a thousand miles. It has taken years to build up and perfect the system and its growth has not yet stopped, nor its importance and value reached its height. It is one of the best examples of co-operative energy that the world affords, nothing equals it in its far-reaching influence or power.

Its accuracy, independence and entire impartiality are to be observed, As to the first, no one, unless he has been inside, knows with what care every piece of news is considered and how the trustworthiness of agents is looked after. There are cases of dismissal where one single word, but an important one, has been overlooked or improperly used. Such are extreme instances, of course, but go to show the diligence and fidelity that are required. Of course the independence of such an immense organization must be apparent on the face of it, although it is not an independence that is regardless of the public, but an independence that prompts it to do just right at the right moment every time, giving what it esteems to be the news, no matter who it may effect. Without its impartiality, its mission would be a failure, for it encompasses within its wide folds, every shade of opinion on every conceivable topic. It could not live and not be impartial.

Descriptions of such enterprises as is the Associated Press, in the view of some, might be likened to talking of the sun-light. Is surrounds us every day, and its constant influence is such that we come to regard it as ordinary, if not common-place, its every day character giving us, quite excusably, the notion that it must be, and that it is not such a wonderful thing after all. But remove it entirely and where would we be? As to the sunlight, philosophers and observers tell us that our earth depends upon it for existence, and analyzing it, find therein, everything that nature needs. Every-day affair as it is, examination proves it a marvel, wonderfully worthy our admiration and gratitude. In its measure likewise is the Associated Press, every-day affair as it is, in its scope, power and value entitled to our admiration for the work it is doing, and to our examination as to the manner in which it is doing it.

